# Performance of lung ultrasonography in children with community-acquired pneumonia

**DOI:** 10.1186/1824-7288-40-37

**Published:** 2014-04-17

**Authors:** Susanna Esposito, Simone Sferrazza Papa, Irene Borzani, Raffaella Pinzani, Caterina Giannitto, Dario Consonni, Nicola Principi

**Affiliations:** 1 Pediatric Highly Intensive Care Unit, Department of Pathophysiology and Transplantation, Università degli Studi di Milano, Fondazione IRCCS Ca’ Granda, Ospedale Maggiore Policlinico, Via Commenda 9, 20122 Milano, Italy; 2 Radiology Unit, Fondazione IRCCS Ca’ Granda, Ospedale Maggiore Policlinico, Milan, Italy; 3 Epidemiology Unit, Fondazione IRCCS Ca’ Granda, Ospedale Maggiore Policlinico, Milan, Italy

**Keywords:** Chest radiography, Community-acquired pneumonia, Lower respiratory tract infection, Lung ultrasonography, Pneumonia, Radiology

## Abstract

**Background:**

There are few prospective evaluations of point-of-care ultrasonography (US) for the diagnosis of pediatric community-acquired pneumonia (CAP). In particular, there are very few data concerning the efficiency of US in comparison with that of chest radiography (CR) in defining different kinds of lung alterations in the various pulmonary sections. The aim of this study was to bridge this gap in order to increase our knowledge of the performance of US in diagnosing CAP in childhood.

**Methods:**

A total of 103 children (56 males, 54.4%; mean age ± standard deviation 5.6 ± 4.6 years) admitted to hospital with a clinical diagnosis of suspected CAP were prospectively enrolled and underwent CR (evaluated by an independent expert radiologist) and lung US (performed by a resident in paediatrics with limited experience in US). The performance of US in diagnosing CAP (i.e. its sensitivity, specificity, and positive and negative predictive values) was compared with that of CR.

**Results:**

A total of 48 patients had radiographically confirmed CAP. The sensitivity, specificity, and positive and negative predictive values of US in comparison with CR were respectively 97.9%, 94.5%, 94.0% and 98.1%. US identified a significantly higher number of cases of pleural effusion, but the concordance of the two methods in identifying the type of CAP was poor.

**Conclusion:**

US can be considered a useful means of diagnosing CAP in children admitted to an Emergency Department with a lower respiratory tract infection, although its usefulness in identifying the type of lung involvement requires further evaluation.

## Background

The incidence of community-acquired pneumonia (CAP) has significantly decreased in places where pneumococcal conjugate vaccines are widely used [1]. However, it is still a very frequent cause of morbidity among children aged ≤5 years living in industrialised countries, where its incidence is about 10–40 cases per 1,000 subjects [2-4]. Most infants and children with CAP present with a number of clinical signs and symptoms, such as fever, cough and tachypnea, and recently published guidelines say that the diagnosis of mild to moderate cases of pediatric CAP can be based solely on clinical criteria without having to be confirmed by chest radiography (CR), which is reserved for severe cases leading to hospital admission or when complications are suspected [5-7]. On the other hand, although CR has some major limitations (including exposure to ionising radiation and a high degree of inter- and intra-observer variability in interpretation) [8], the absence of CR confirmation leads to the incidence of CAP being largely overestimated and causes a number of problems, the most important of which is an increased use of unnecessary antibiotics.

In an attempt to overcome this problem, the use of ultrasonography (US) was suggested in 1986, when Weinberg *et al*. described a new method of evaluating CAP by means of the demonstration of sonographic air bronchograms within lung consolidations [9]. However, although US is considered to be reliable and safe, its use was long confined to a supplementary role in the case of complex CAP, mainly because there were no portable or hand-held machines. It is only recently that technological advances have allowed these difficulties to be overcome and revived interest in using US to diagnose lower respiratory tract infections. A number of studies have shown that it is feasible and accurate in diagnosing lung infections when used by experienced clinician-sonologists [10-17], but there are few prospective evaluations of point-of-care US for the diagnosis of pediatric CAP. In particular, there are very few data concerning the efficiency of US in comparison with that of CR in defining different kinds of lung alterations in the various pulmonary sections.

The aim of this study was to bridge this gap in order to increase our knowledge of the performance of US in diagnosing CAP.

## Methods

### Study design and patients

This prospective observational study was carried out in Pediatric Highly Intensive Care Unit of the Department of Pathophysiology and Transplantation of the University of Milan between 1 November 2012 and 28 February 2013. It was approved by the Ethics Committee of Milan’s Fondazione IRCCS Ca’ Granda, Ospedale Maggiore Policlinico, and required the written informed consent of a parent or legal guardian; the older children were asked to give their assent.

All of the otherwise healthy children born at term aged between 1 month and 14 years admitted with fever (i.e. an axillary temperature of >38°C) and signs and symptoms consistent with CAP (i.e. cough, tachypnea, dyspnea or respiratory distress, and breathing with grunting or wheezing sounds with rales) and hospitalized in our pediatric ward were considered eligible for inclusion. The patients who arrived in the Emergency Department with a chest radiograph previously taken in another hospital and those with hemodynamic instability were excluded.

### Radiological examinations

Upon enrolment, all of the children with suspected CAP underwent CR, both postero-anterior and lateral views as recommended in recently published guidelines [6]. The radiographs were evaluated by an independent expert radiologist (IB), who classified the findings as alveolar pneumonia, non-alveolar (interstitial) pneumonia, mixed interstitial and alveolar pneumonia, or no pneumonia in accordance with the World Health Organisation (WHO) criteria for the standardised interpretation of pediatric chest radiographs for the diagnosis of pneumonia [18]. Immediately after CR, in all of the patients lung US evaluation of the lungs was performed.

The physician performing lung US was always the same (SSP), was a resident in paediatrics and had limited experience in US prior to the study. He had no contact with the radiologist, and was blinded to the CR findings. Before starting the study, he underwent a one-day lung US training session (given by IB) consisting of a 3-hour lecture on lung US (including instructions on differentiating dynamic from static air bronchograms as well as lung US findings consistent with bronchiolitis in children under 2 years of age) and the recognition of disease and potential errors, followed by a 4-hour practical hands-on imaging session of normal models. The lung US was carried out using a MyLab™25 Gold (Esaote, Genoa, Italy) with a convex 2.5-6.6 MHz probe and a linear 7.5-12 MHz probe. The probes were placed perpendicularly, obliquely and parallel to the ribs in the anterior, lateral and posterior thorax (lower and upper) as described by Copetti and Cattarossi [19], and were moved longitudinally and transversely. The posterior thorax was scanned with the patients in lateral decubitus and sitting positions. Alveolar CAP was diagnosed in the presence of lung consolidation with sonographic air bronchograms, whereas interstitial CAP was diagnosed in the presence of B lines or an interruption of the normal course of the pleural line (superficial fluid alveologram). Mixed CAP was diagnosed in the presence of data consistent with both alveolar and interstitial CAP in the same patient. The presence of pleural effusion was also evaluated in detail.

### Statistical analysis

The performance of US in diagnosing CAP (i.e. its sensitivity, specificity, and positive and negative predictive values) was compared with that of CR, considered the gold standard for CAP diagnosis in children according to published guidelines [5-7]. The concordance of US and CR in identifying specific types of CAP (i.e. alveolar, mixed, non-alveolar) and pleural effusion was evaluated using Cohen’s weighted kappa (*k*) statistics [20]. The discrepancies between US and CR were assessed using marginal homogeneity Stuart-Maxwell statistics [21,22], which are analogous to the McNemar chi-squared test for paired data but can also be used for variables with more than two categories. The analyses were made using Stata 13 software (Stata Corp., 2013).

## Results

Forty-eight of the 103 enrolled children (56 males, 54.4%; mean age ± standard deviation [SD] 5.6 ± 4.6 years) had radiographically confirmed CAP. Table 1 shows the sensitivity, specificity, and positive and negative predictive values of US in comparison with CR. All of the studied variables were higher than 94%. CR detected one case of CAP that was missed by US, whereas there were three cases with sonographic air bronchograms <1 cm detected at US, that were negative at CR. These three cases showed air bronchograms at US in the same lobe where physical examination revealed rales at the auscultation.

**Table 1:** **Sensitivity (Se), specificity (Sp), positive predictive value (PPV), and negative predictive value (NPV) of lung ultrasonography (US) *vs *chest radiography (CR) in diagnosing community- acquired pneumonia**

	**Lung US**		**Se (%)**	**Sp (%)**	**PPV (%)**	**NPV (%)**
**CR**	**Negative**	**Positive**	**(95% CI)**	**(95% CI)**	**(95% CI)**	**(95% CI)**
**Negative**	52	3	97.9	94.5	94.0	98.1
**Positive**	1	47	(88.9-99.9)	(84.9-98.9)	(83.5-98.7)	(89.9-100)

*CI* confidence interval.

Table 2 shows the CR and US results in diagnosing specific types of CAP. The concordance between the two methods was relatively good (*k* = 0.64). However, beyond the three US-positive/CR-negative cases mentioned above, there were 17 cases with non-alveolar CAP at CR that were diagnosed as mixed (4 cases) or alveolar (13 cases) using US. In addition, 5 mixed CAP cases were diagnosed as alveolar by US. In general, US tender to overdiagnose CAP or to err on the side of diagnosing an alveolar CAP (25 cases in the upper right off-diagonal cells vs 3 cases in the lower left cells, p = 0.0009 at the marginal homeogeneity Stuart-Maxwell test).

**Table 2:** Comparison of chest radiography (CR) and lung ultrasonography (US) results in diagnosing specific types of community-acquired pneumonia

	**Lung US**				
**CR**	**Negative**	**Interstitial**	**Mixed**	**Alveolar**	**Total**
**Negative**	**52**	-	3	-	55
**Interstitial**	-	**8**	4	13	25
**Mixed**	1	-	**6**	5	12
**Alveolar**	-	1	1	**9**	11
**Total**	53	9	14	27	103

Weighted Cohen’s kappa: 0.64 (p < 0.0001).

Marginal homogeneity (Stuart-Maxwell) test: p = 0.0009.

Concordant results in bold.

Table 3 shows the CR and US results in diagnosing specific types of CAP in different lung districts. Concordance was moderate to good when evaluated with the *k* statistics, but again in all of the studied anatomical districts the type of lung lesion was differently identified by US and CR.

**Table 3:** Comparison of chest radiography (CR) and lung ultrasonography (US) results in diagnosing specific types of community-acquired pneumonia (CAP) in different anatomical districts

**Right lung, upper**	k = 0.54 (p < 0.0001)	MH-SM p = 0.046			
	**Lung US**				
**CR**	**Negative**	**Interstitial**	**Mixed**	**Alveolar**	**Total**
**Negative**	**75**	3	2	-	80
**Interstitial**	5	**5**	2	1	13
**Mixed**	-	-	**2**	-	2
**Alveolar**	2	3	1	**2**	8
**Total**	82	11	7	3	103
**Right lung, lower**	k = 0.59 (p < 0.0001)	MH-SM p = 0.002			
	**Lung US**				
**CR**	**Negative**	**Interstitial**	**Mixed**	**Alveolar**	**Total**
**Negative**	**65**	1	1	1	68
**Interstitial**	7	**4**	1	0	12
**Mixed**	-	-	**4**	-	4
**Alveolar**	6	2	5	**6**	19
**Total**	78	7	11	7	103
**Left lung, upper**	k = 0.55 (p < 0.0001)	MH-SMp = 0.20			
	**Lung US**				
**CR**	**Negative**	**Interstitial**	**Mixed**	**Alveolar**	**Total**
**Negative**	**82**	6	1	1	90
**Interstitial**	3	**7**	1	-	11
**Mixed**	-	-	**1**	-	1
**Alveolar**	-	-	1	**-**	1
**Total**	85	13	4	1	103
**Left lung, lower**	k = 0.63 (p < 0.0001)	MH-SM p = 0.03			
	**Lung US**				
**CR**	**Negative**	**Interstitial**	**Mixed**	**Alveolar**	**Total**
**Negative**	**68**	2	1	1	72
**Interstitial**	5	**5**	1	1	12
**Mixed**	-	-	**2**	-	2
**Alveolar**	4	2	4	**7**	17
**Total**	77	9	8	9	103

*MH-SM* marginal homogeneity (Stuart-Maxwell) test.

Concordant pairs in bold.

Table 4 shows the CR and US results in diagnosing pleural effusion. US identified a significantly higher number of cases in both the right and left pleural space.

**Table 4:** Comparison of chest radiography (CR) and lung ultrasonography (US) results in diagnosing pleural effusion

**Pleural effusion, right**	k = 0.30 (p < 0.0001)	MH-SM p = 0.0005	
	**Lung US**		
**CR**	**Absent**	**Present**	**Total**
**Absent**	**88**	12	100
**Present**	0	**3**	3
**Total**	88	15	103
**Pleural effusion, left**	k = 0.16 (p = 0.02)	MH-SM p = 0.004	
	**Lung US**		
**CR**	**Absent**	**Present**	**Total**
**Absent**	**86**	13	99
**Present**	2	**2**	4
**Total**	88	15	103

*MH-SM* marginal homogeneity (Stuart-Maxwell) test.

Concordant pairs in bold.

## Discussion

The findings of this study confirm that lung US is a simple and reliable imaging technique that is nearly as reliable as CR in identifying the lung lesions that are diagnostic for CAP, and also show that it is even more effective than CR in diagnosing pleural effusion [10-17]. These data further support the recent recommendation of the International Liaison Committee on Lung Ultrasound to use US in pediatric patients with suspected CAP in order to reduce antibiotic abuse [15].

US has a number of advantages: it is easy to do at a child’s bedside, takes little time to perform and interpret the results, allows a close follow-up, and avoids the use of ionising radiation, which is particularly important in pediatrics because children are at least four times more sensitive than adults to the induction of cancer [23]. It has also been recently shown that CR frequently identifies acute bronchitis in the presence of signs and symptoms of mild to moderate CAP [24,25]. As antibiotic treatment is not recommended for children diagnosed as having bronchitis [5-7], the use of lung US in children with clinical findings suggesting mild-to-moderate CAP could reduce the abuse of antibiotics, thus overcoming one of the limitations of CR. Furthermore, the lung US examinations in our study were performed by a non-radiologist clinician after only a short period of training, and the good results in terms of overall efficiency in comparison with CR highlight the fact that lung US could be easily used in the outpatient setting to reduce antibiotic abuse and CAP-associated costs, although further studies specifically performed in the pediatric outpatient setting are needed.

However, the concordance of the US and CR data was relatively limited in terms of the type of lung lesions revealed, and this may have important implications when it comes to prescribing antibiotics. A considerable number of cases were defined alveolar CAP by US but non-alveolar CAP by CR. In the traditional recommendations, most cases of alveolar CAP are considered to be due to a typical bacterial infection requiring beta-lactam antibiotic therapy, whereas interstitial CAP is thought to be mainly due to viruses that do not require antibiotics or atypical bacteria that require macrolides [5-7]. This discordant result may have been due to the different limit for the CR detection of lung consolidation. Shah *et al*. have reported that lung consolidations of ≤1 cm are undetectable by CR, which remains negative or suggests moderate infiltration resembling non-alveolar disease [17]. There were similar significant differences when the site of lung damage suggesting CAP was evaluated: the concordance of the two methods was only moderate, thus confirming the difficulty of identifying lung damage by US regardless of the type of CAP. Although this result may have been affected by the experience of the physician performing the lung US, further data are needed to confirm the role of US in identifying the possible etiology of CAP and studying the characteristics of lung involvement and their correlations also with clinical findings.

Regarding the diagnosis of pleural effusion, US appeared better than CR, but comparison has not been done with CT scan which represents the gold standard for its evaluation [26-28]. However, in comparison with CT, US does not use inonising radiation and has significantly lower costs.

Although this study highlights new possibilities in diagnostic approach to pediatric CAP, it has some limits. First of all, all patients were hospitalized, and no evaluation on children managed in the outpatient setting is available. Moreover, despite a pre-study analysis has been performed on the optimal sample size of patients and significant differences have been observed, the number of radiologically-confirmed CAP cases is not too high in order to draw a definitive judgment on US’s utility. This is also the reason for which the correlation between US findings and laboratory data has not been done, but further researches should evaluate this correlation. Furthermore, the present research is not a multicentric study and further studies should consider the concordance between operators in performing the lung US examinations.

## Conclusion

US can be considered a useful means of diagnosing CAP in all children admitted to an Emergency Department with lower respiratory tract infection. However, its usefulness in identifying the type of lung involvement is uncertain, could be influenced by the operator’s learning curve, and requires further evaluation before US can be considered reliable enough to be used for making decisions concerning antibiotic treatment in children with CAP.

## Abbreviations

CAP: Community-acquired pneumonia; CI: Confidence interval; CR: Chest radiography; MH-SM: Marginal homogeneity (Stuart-Maxwell) test; NPV: Negative predictive value; OR: Odds ratio; PPV: Positive predictive value; SD: Standard deviation; Se: Sensitivity; Sp: Specificity; US: Ultrasonography.

## Competing interests

The authors declare that they have no competing interests.

## Authors’ contributions

SE designed the study, coordinated the project and drafted the manuscript; SSP performed lung US; IB performed and evaluated all the CRs; RF enrolled the patients and followed-up them clinically; CG contributed to the revision of lung US; DC performed the statistical analysis; NP supervised the project and co-wrote the draft manuscript. All of the authors read and approved the final version of the manuscript.
